# Characteristics of and changes in the cardiometabolic measures of Japanese workers grouped according to their vegetables and salt intake through workplace cafeteria meals

**DOI:** 10.1017/S1368980024001162

**Published:** 2024-05-24

**Authors:** Yoshiro Shirai, Masae Sakuma, Yusuke Ushida, Takayuki Imoto, Keisuke Suga, Kunio Matsui, Mieko Nakamura

**Affiliations:** 1 Department of Food and Nutritional Environment, Kinjo Gakuin University, Nagoya, Aichi, Japan; 2 Department of Food and Health Sciences, International College of Arts and Sciences, Fukuoka Women’s University, Fukuoka, Japan; 3 Innovation Division, Kagome Co., Ltd, Nagoya, Aichi, Japan; 4 Safety & Health Promotion Division, Toyota Motor Corporation, Toyota, Aichi, Japan; 5 Agriculture & Biotechnology Business Division, Toyota Motor Corporation, Toyota, Aichi, Japan; 6 Department of Community Health and Preventive Medicine, Hamamatsu University School of Medicine, Hamamatsu, Shizuoka, Japan

**Keywords:** Vegetable intake, Salt intake, Occupational health, Workplace cafeteria, CVD risk

## Abstract

**Objective:**

This study aimed to objectively evaluate the diet consumed in a workplace cafeteria to group Japanese workers according to vegetables and salt intake and estimate the association of these groups with changes in cardiometabolic measurements.

**Design:**

This longitudinal observational study estimated the food and nutrient intake of Japanese workers from data recorded in the cafeteria system of their workplace. The primary outcomes included cardiometabolic measures obtained via regular health check-ups conducted at the workplace. The participants were divided into four groups according to high or low vegetables and salt intake based on their respective medians, and the association of each group with cardiometabolic measurement changes was estimated using robust regression with MM-estimation.

**Setting:**

A Japanese automobile manufacturing factory.

**Subjects:**

The study included 1140 men and women workers with available cafeteria and health check-up data.

**Results:**

An inverse marginal association was observed between changes in TAG levels (mmol/L) and high vegetables and low salt intake (*β*: –0·11, 95 % CI: –0·23, 0·01, *P*: 0·065) with reference to low vegetables and high salt intake. This association was stronger in participants who used the cafeteria more frequently (>=71 d; *β*: –0·15, 95 % CI: –0·29, –0·02, *P*: 0·027).

**Conclusions:**

The participants in the higher vegetables and lower salt intake group were more likely to exhibit decreased TAG levels. These findings encourage using workplace cafeteria meals to promote the health of workers.

Ensuring the health of workers is a key foundation for a sustainable society. As a corporate health promotion strategy, dietary management in workplace cafeterias is considered important for the well-being of the workforce^(1,2)^. The International Labour Organization (ILO) reports that the global burden of diet-related diseases is increasing because of the enormous costs of chronic disease and obesity. As such, the ILO recommends the provision of healthy meals to workers in the workplace^(3)^. In addition, the scientific community considers diet and nutrition important for the health management of workers in the workplace, regarding which several intervention studies have been conducted^(4)^. However, few studies have investigated the actual meals that workers consume in workplace cafeterias and the association between such meals and health parameters.

Considerable evidence indicates that vegetables play a fundamental role in a healthy diet. Moreover, obesity, hypertension, hyperglycaemia and cholesterol abnormalities, which can result from poor dietary habits, are reportedly strongly correlated with total medical, worker compensation and short-term disability costs^(5)^. Thus, an actionable approach to these classical CVD risk factors is important to ensure occupational health. Although the findings of meta-analyses suggest that vegetables intake improves cardiometabolic factors^(6–9)^, reports regarding the association between actual vegetables intake in workplace cafeterias and cardiometabolic factors are lacking.

Vegetables are an essential part of a healthy diet; however, the same is not true regarding excessive salt intake. The WHO has issued guidelines for developing national policies and public health nutrition programmes that recommend adequate Na intake alongside guidelines for the intake of potassium and other nutrients to reduce noncommunicable diseases^(10)^. According to a survey by the Japanese Ministry of Health, Labour and Welfare, the average daily salt intake for Japanese men and women is 10·9 and 9·3 g, respectively, which is excessive, whereas the daily vegetables intake is approximately 290 for men and 270 g for women, which is less than the 350 g recommended in the Japanese Food Guide Spinning Top^(11)^. However, a positive association between vegetables and salt intake has also been reported^(12–14)^, and dietary habits promoting more vegetables and less salt consumption are important. Several randomised controlled trials and observational studies have suggested that potassium supplementation, of which vegetables are the main source, attenuates the influence of Na on elevated blood pressure^(15–18)^. Accordingly, previous studies have assessed the effects of both Na and potassium on cardiovascular health, reporting a higher risk of death from CVD when Na intake is high relative to that of potassium^(19)^. This signifies that salt intake should also be assessed in combination with vegetables intake. The current study aimed to provide basic information regarding workplace cafeteria meals to address the issue of inadequate vegetables and excessive salt intake in Japan.

Herein, data that were automatically collected by a payment system using tableware with an integrated-circuit chip embedded with menu information and employee identification cards enabled the objective evaluation of the meals consumed in the real-life setting of a workplace cafeteria. This study described the characteristics of dietary, anthropometric, lifestyle and socio-economic factors related to vegetables and salt intake and estimated the association between the groups stratified by actual vegetables and salt intake in the workplace cafeteria and cardiometabolic health.

## Methods

### Study participants

The study sample was Japanese workers in the automobile manufacturing industry who utilized workplace cafeterias that were open during the designated break times in each shift. Approximately 1821 of 2069 employees had meals in the cafeteria during the period from which data were accessible (i.e. 1 July 2019 to 30 September 2020). Those who used the cafeteria less than three times during the period (*n* 136) or those who did not have any data from two or more health examinations (*n* 545) were excluded. Finally, 1140 individuals with available data were selected for the present analysis.

### Dietary survey

The eating habits of the participants were estimated from the daily use data accumulated in the electronic purchase system of the cafeteria. Details regarding the electronic purchase system and the calculation of nutrient intakes have been reported elsewhere^(20)^. Briefly, the tableware used in the cafeteria had an integrated-circuit chip embedded with menu information; after users consumed their meal, the chip information was read by a dedicated reader, and the users paid for the selected menu item using their employee identification card. Through this operation, data concerning the daily selected per-use meals by the workers were objectively and automatically recorded in the system on a serving basis. Foods were classified into the following fourteen food groups according to the classification in the Standard Tables of Food Composition in Japan – 2015 Edition (7th Revision)^(21)^: ‘cereals’, ‘potatoes and starches (hereafter potatoes)’, ‘sugars and sweeteners (hereafter sugars)’, ‘pulses’, ‘nuts and seeds (hereafter nuts)’, ‘vegetables’, ‘fruits’, ‘mushrooms’, ‘algae’, ‘fishes and shellfishes (hereafter fishes)’, ‘meats’, ‘eggs’, ‘milks’ and ‘fats and oils’. According to the Japanese Food Guide Spinning Top^(22)^, the dishes were categorised into the following groups: grain dishes (syushoku: staple dishes), including rice, bread, noodles and pasta, wherein the main ingredient is a source of carbohydrates; fish and meat dishes (syusai: main dishes), including meat, fish, egg and soyabean dishes, wherein the main ingredient is a source of protein; vegetable dishes (fukusai: side dishes), including vegetables, potatoes, pulses (excluding soya), mushrooms and algae, wherein the main ingredient is a source of various vitamins, minerals and dietary fibre. Examples of the food composition of dishes actually served in the cafeteria during the study period are shown in the online supplementary material, Supplemental Table S1. Furthermore, the cafeteria served healthy meal sets with >140 g vegetables and <3·0 g salt, that is, vegetable-rich and low-salt meals prepared by registered dietitians who designed the foods and cooking methods. Herein, the average of at least three dietary records was used as the habitual food group and nutrient intake of the participants. Therefore, the definition of the amount of food consumed was the serving amount, whereas the amount of leftover food per individual could not be assessed. Regarding salt in the noodle broth, the salt intake was estimated by referring to a previous report of salt intake from broth and the broth intake when noodles were consumed by Japanese individuals^(23)^ alongside the assumption that only half the broth was consumed.

### Health examination data

Details regarding the measurement instruments and sample assays for the health examination data have been described elsewhere^(20)^. Briefly, the study participants received regular health check-ups at in-house company sites about the same month each year (approximately 150–200 people/month). The health examination included anthropometric measurements (BMI, systolic blood pressure and diastolic blood pressure), blood tests (TAG, LDL-cholesterol (LDL-C), HDL-cholesterol (HDL-C) and Hb A1c (HbA1c)), self-administered questionnaires (exercise, drinking and smoking habits) and interviews (hospital visits and medication). Blood tests were performed after fasting for at least 10 h. However, for employees aged <35 years, the blood tests were conducted only once every 2 years. The study used data from health examinations of those with at least two records between 1 October 2018 and 30 September 2020, that is, the period when data were accessible; data from the first record were used as the baseline data, and data from the last record were used as the follow-up data. TGA levels were converted from mg/dL to mmol/L by multiplying by 0.0113, and LDL-C and HDL-C levels were converted by multiplying by 0.0259.

### Statistical analysis

Food group and nutrient intake were adjusted for the total energy intake using the residual method. The participants were divided into four vegetables–salt groups according to the median intake of vegetables (105·7 g) and salt (4·0 g): low vegetables/low salt intake (VL_SL; *n* 395), low vegetables/high salt intake (VL_SH; *n* 175), high vegetables/low salt intake (VH_SL; *n* 175) and high vegetables/high salt intake (VH_SH; *n* 395). The associations of each vegetable and salt intake with baseline dietary, anthropometric and blood tests and lifestyle and socio-economic factors were analysed using the generalised linear model. Likewise, the associations between the four vegetables–salt groups and those factors were analysed. In the generalised linear model, Gaussian, Poisson and binary were specified as link functions depending on the distribution of the objective variables. The baseline values for TAG, LDL-C and HDL-C were natural log transformed.

The outcome of cardiometabolic factors was defined as the change calculated by subtracting the baseline value from the follow-up value. Robust regression with MM-estimation was used to de-emphasise the outliers of objective and explanatory variables. The procedure uses an iteratively reweighted least squares approach to weight each data point based on the magnitude of its residuals, with larger residuals being assigned smaller weights. The objective variable was the change in cardiometabolic measurements, and the explanatory variable was a dummy variable for the four vegetables–salt groups referencing the VL_SH group. We estimated the association between the change in the cardiometabolic measurements and four vegetables–salt groups, controlling for adjustment variables included in the following three models: Model 1, adjusted for age, sex and BMI (not included if the objective variable was BMI); Model 2, added to Model 1 for exercise, alcohol, smoking, total energy intake, frequency of cafeteria visit and medication (antihypertensive medicine for systolic blood pressure and diastolic blood pressure; antilipidemic medicine for LDL-C, HDL-C and TAG; and antidiabetic medicine for HbA1c); and Model 3, added to Model 2 for the baseline values of the objective variable.

Only men (*n* 1089) were included in the sensitivity analysis. A sensitivity analysis was also performed among those who had used the cafeteria >=18 times (*n* 1027) or >=71 d (*n* 963). These estimates were based on the minimum number of days of data collection required to estimate the true vegetables intake of an individual within 20 and 10 % of their true mean value, respectively^(24)^. Online supplementary material, Supplemental Fig. S1, shows the distribution of the frequency of cafeteria use during the study period. Furthermore, with respect to the model, sensitivity analyses were conducted on models with additional adjustment variables, including sedentary time, weight measurement, occupation, company position, work shift, working hours, breakfast intake frequency, subjective eating speed and time between dinner and sleep.

All analyses were performed using R 4·2·2, and generalised linear models were fitted using the glm function of the STATS package^(25)^. In the robust regression models, coefficients were estimated using the rlm function in the MASS package^(26)^, while the 95 % CI were estimated using the model_parameters function in the parameters package^(27)^.

## Results

### Characteristics of participants grouped according to their vegetables and salt intake

The characteristics of participants according to the four vegetables–salt groups are described in Table 1. The participants used the cafeteria a mean (sd) total of 198·5 (92·7) times during the study period, and their total energy intake per use was 814·8 (149·7) kcal. The mean vegetables intake per use for the VL_SL, VL_SH, VH_SL and VH_SH groups was 85·1 (12·3), 90·0 (12·3), 124·5 (19·4) and 130·8 (21·6) g, respectively, with slightly higher vegetables intake for the high salt intake groups compared with that in the low salt intake groups. Similarly, the mean salt intake per use for the VL_SL, VL_SH, VH_SL and VH_SH groups was 3·6 (0·3), 4·3 (0·3), 3·7 (0·2) and 4·5 (0·4) g, respectively, with a slightly higher mean salt intake among the high vegetables intake groups (i.e. VH_SL and VH_SH) compared with that in the low vegetables intake groups (i.e. VL_SL and VL_SH).

**Table 1.** Characteristics of cafeteria usage records between 1 July 2019 and 30 September 2020 according to vegetables/salt intake group

	VL_SL			VL_SH (Ref)			VH_SL			VH_SH			All		
Variables	*n* 395			*n* 175			*n* 175			*n* 395			*n* 1140		
Cafeteria visit, total times	200·5	(93·6)		199·7	(93·5)		190·2	(99·4)		199·8	(88·5)		198·5	(92·7)	
Total energy, kcal^†^	815·3	(141·0)		797·8	(155·1)		791·9	(160·2)		832·2^*^	(149·3)		814·8	(149·7)	
Grain dishes, times^†^	1·14.	(0·2)		1·18	(0·3)		1·07^***^	(0·2)		1·05^***^	(0·2)		1·11	(0·2)	
Fish and meat dishes, times^†^	0·71^***^	(0·3)		0·85	(0·3)		0·76^**^	(0·3)		0·91^*^	(0·3)		0·81	(0·3)	
Vegetable dishes, times^†^	0·62	(0·4)		0·65	(0·4)		0·99^***^	(0·3)		1·13^***^	(0·5)		0·86	(0·5)	
Healthy meal sets^‡^, times^†^	0·01	(0·0)		0·01	(0·0)		0·16	(0·3)		0·07^***^	(0·2)		0·05^***^	(0·2)	
Cereals, g^†^,^§^	168·7^***^	(44·5)		191·2	(51·6)		147·5^***^	(36·5)		152·7^***^	(40·1)		163·4	(45·4)	
Potatoes, g^†^,^§^	11·4	(5·2)		11·4	(5·9)		15·0^***^	(6·4)		16·7^***^	(6·8)		13·8	(6·6)	
Sugars, g^†^,^§^	1·6^**^	(0·6)		1·7	(0·8)		1·6	(0·8)		1·8	(0·7)		1·7	(0·7)	
Pulses, g^†^,^§^	16·1^***^	(13·2)		33·5	(30·2)		16·4^***^	(12·5)		26·8^***^	(22·8)		22·5	(21·2)	
Nuts, g^†^,^§^	0·4	(0·3)		0·4	(0·3)		0·4	(0·3)		0·5	(0·3)		0·4	(0·3)	
Vegetables, g^†,§^	85·1^**^	(12·3)		90·0	(12·3)		124·5^***^	(19·4)		130·8^***^	(21·6)		107·7	(27·3)	
Fruits, g^†^,^§^	4·2^*^	(5·0)		3·2	(3·9)		3·2	(4·7)		2·7	(3·7)		3·4	(4·4)	
Mushrooms, g^†^,^§^	1·3	(0·9)		1·5	(1·0)		2·3^***^	(1·6)		2·3^***^	(1·4)		1·8	(1·3)	
Algae, g^†^,^§^	0·7^***^	(0·4)		0·9	(0·6)		0·8	(0·5)		1·0	(0·6)		0·8	(0·5)	
Fishes, g^†^,^§^	11·6^***^	(7·7)		15·9	(10·7)		12·6^**^	(6·9)		19·0^**^	(12·2)		15·0	(10·4)	
Meats, g^†^,^§^	98·0	(49·3)		105·3	(58·3)		88·6^***^	(32·2)		91·5^**^	(36·5)		95·4	(44·8)	
Eggs, g^†^,^§^	17·4	(8·4)		18·5	(9·3)		16·4^*^	(8·0)		18·7	(8·4)		17·9	(8·5)	
Milks, g^†^,^§^	6·0^*^	(6·7)		4·7	(5·6)		4·7	(5·8)		4·0	(4·9)		4·9	(5·8)	
Fats and oils, g^†^,^§^	9·3^**^	(2·6)		8·5	(2·6)		9·4^**^	(2·4)		9·0^*^	(2·3)		9·1	(2·5)	
Salt, g^†^,^§^	3·6^***^	(0·3)		4·3	(0·3)		3·7^***^	(0·2)		4·5^***^	(0·4)		4·0	(0·5)	
Protein, g^†^,^§^	28·2^***^	(2·2)		30·3	(2·7)		28·7^***^	(2·4)		30·7^*^	(2·7)		29·5	(2·7)	
Fat, g^†^,^§^	28·6	(3·6)		28·5	(3·9)		29·3^*^	(3·0)		30·7^***^	(3·8)		29·4	(3·8)	
Carbohydrate, g^†^,^§^	109·6	(9·2)		108·6	(10·0)		107·8	(7·9)		103·7^***^	(10·0)		107·1	(9·7)	

Values are presented as mean (sd).

VL_SL, low vegetables intake and low salt intake; VL_SH, low vegetables intake and high salt intake; VH_SL, high vegetables intake and low salt intake; VH_SH, high vegetables intake and high salt intake.

* *P* < 0·05.

** *P* < 0·01.

*** *P* < 0·001; general linear model.

† Average per cafeteria visit.

‡ Healthy meal sets include a grain, fish and meat and vegetable dishes and contain <3·0 g of salt and >140 g of vegetables.

§ Energy adjusted value by the residual method.

Anthropometric and blood test data of the four vegetables–salt group are described in Table 2. The mean (sd) age of participants was 39·4 (11·5) years, with a higher mean age in the high vegetables intake groups compared with that in the low vegetables intake groups. Men accounted for 96 % of the participants. Regarding the baseline cardiometabolic factors, the mean (sd) BMI was 22·9 (3·5) kg/m^2^, systolic blood pressure was 120·6 (12·5) mmHg, diastolic blood pressure was 71·8 (11·0) mmHg, TAG was 1·27 (1·17) mmol/L, LDL-C was 2·98 (0·77) mmol/L, HDL-C was 1·56 (0·38) mmol/L, and HbA1c was 5·4 (0·5) % for the entire study population. The mean baseline measurements of these cardiometabolic factors (including HDL-C) were higher in the high vegetables intake groups compared with that in the low vegetables intake groups, with the exception of TAG in the VL_SH group, which had a higher mean and sd because of one individual with very high values. Similarly, the high vegetables intake group also had a higher proportion of participants with high-risk cardiometabolic health at the baseline health examination compared with that in the low vegetables intake groups.

**Table 2.** Distribution of anthropometric and blood test measurements according to vegetables/salt intake group^†^

	VL_SL				VL_SH (Ref)				VH_SL				VH_SH				All			
Variables	*n*	Mean (sd)/%			*n*	Mean (sd)/%			*n*	Mean (sd)/%			n	Mean (sd)/%			n	Mean (sd)/%		
Age (year)	395	36·1^**^	(10·7)		175	39·5	(11·6)		175	41·3	(12·0)		395	41·8^*^	(11·3)		1140	39·4	(11·5)	
Men	375	95·0			171	98·0			169	97·0			374	95·0			1089	96·0		
BMI, kg/m^2^	395	22·1	(3·4)		175	22·6	(2·8)		175	23·0	(3·4)		395	23·8^***^	(3·6)		1140	22·9	(3·5)	
SBP, mmHg	395	119·0	(11·8)		175	119·3	(13·4)		175	122·1^*^	(13·2)		395	122·0^*^	(12·2)		1140	120·6	(12·5)	
DBP, mmHg	395	69·8	(11·1)		175	70·6	(11·0)		175	73·4^*^	(10·7)		395	73·7^**^	(10·7)		1140	71·8	(11·0)	
TAG, mmol/L	333	1·18^*^	(0·94)		150	1·42	(1·89 )		153	1·29	(0·95)		358	1·28	(1·03)		994	1·27	(1·17)	
LDL-C, mmol/L	333	2·87	(0·77)		150	2·94	(0·78)		153	3·04	(0·82)		358	3·08	(0·75)		994	2·98	(0·78)	
HDL-C, mmol/L	333	1·58	(0·37)		150	1·51	(0·37)		153	1·61^*^	(0·41)		358	1·53	(0·38)		994	1·56	(0·38)	
HbA1c	327	5·3	(0·4)		149	5·3	(0·4)		149	5·4^*^	(0·5)		353	5·5^**^	(0·6)		978	5·4	(0·5)	
BMI >= 25 kg/m^2^	76	19·0			38	22·0			32	18·0			120	30·0^***^			266	23·0		
SBP >= 130 or DBP >= 85 mmHg	91	23·0			39	22·0			54	31·0			120	30·0^*^			304	27·0		
LDL-C >= 3·11 mmol/L	124	37·0			61	41·0			74	48·0^*^			178	50·0^***^			437	44·0		
HDL-C =1·04 mmol/L	12	3·6			9	6·0			6	3·9			19	5·3			46	4·6		
TAG >= 1·70 mmol/L	49	15·0			28	19·0			33	22·0			66	18·0			176	18·0		
HbA1c >= 5·6	57	17·0			27	18·0			38	26·0^*^			108	31·0^***^			230	24·0		
Change in BMI, kg/m^2^	395	0·3	(0·8)		175	0·1	(0·9)		175	0·2	(0·9)		395	0·2	(0·9)		1140	0·2	(0·9)	
Change in SBP, mmHg	395	1·2	(11·3)		175	2·0	(13·1)		175	1·8	(11·4)		395	1·2	(11·5)		1140	1·4	(11·7)	
Change in DBP, mmHg	395	0·2	(8·6)		175	0·3	(8·7)		175	–0·5	(8·2)		395	–0·4	(8·9)		1140	–0·1	(8·6)	
Change in TAG, mmol/L	262	0·02	(0·73)		131	–0·15	(1·92)		135	–0·03	(0·97)		315	0·02	(0·87)		843	–0·01	(1·08)	
Change in LDL-C, mmol/L	262	0·06	(0·47)		131	0·05	(0·60)		135	–0·08^*^	(0·49)		315	0·02	(0·52)		843	0·02	(0·51)	
Change in HDL-C, mmol/L	262	0·02	(0·20)		131	0·04	(0·20)		135	0·003	(0·2)		315	0·02	(0·22)		843	0·02	(0·21)	
Change in HbA1c	245	–0·05	(0·3)		124	–0·02	(0·2)		128	–0·04	(0·3)		301	–0·06	(0·3)		798	0·05	(0·3)	

VL_SL, low vegetables intake and low salt intake; VL_SH, low vegetables intake and high salt intake; VH_SL, high vegetables intake and low salt intake; VH_SH, high vegetables intake and high salt intake; SBP, systolic blood pressure; DBP, diastolic blood pressure; LDL-C, LDL-cholesterol; HDL-C, HDL-cholesterol; HbA1c, Hb A1c.

* *P* < 0·05.

** *P* < 0·01.

*** *P* < 0·001; generalised linear model (Gaussian or binomial distribution).

† Baseline data and changes up to the end of follow-up shown.

Data related to the lifestyle and socio-economic factors of the workers according to the vegetables–salt groups are described in Table 3. Among the entire study population, the percentages of those who exercised at least once a week, drank alcohol and smoked were 71·3, 74·0 and 34·4 %, and the percentages of those who took antidiabetic, antihypertensive or antilipidemic medications were 2·8, 8·9 and 7·5 %, respectively.

**Table 3.** Distribution of lifestyle and socio-economic factors according to vegetables/salt intake group

	VL_SL		VL_SH (Ref)		VH_SL		VH_SH		All	
Variables	n	%	n	%	n	%	n	%	n	%
Lifestyle										
Exercise		^*^						^*^		
>= 1 times a week	262	66·3	134	76·6	121	69·1	296	74·9	813	71·3
Alcohol						^*^				
Drinker	278	70·4	122	69·7	139	79·4	299	75·7	838	74·0
Smoking						^***^		^***^		
Current smoker	154	39·0	80	45·7	43	24·6	115	29·1	392	34·4
Breakfast										
Do not eat	18	4·6	2	1·1	3	1·7	10	2·5	33	2·9
Eat occasionally	100	25·3	32	18·3	19	10·9	79	20·0	230	20·2
Eat every day	277	70·1	141	80·6	153	87·4	306	77·5	877	76·9
Subjective eating speed										
Slow	48	12·2	15	8·6	14	8·0	35	8·9	112	9·8
Normal	236	59·7	103	58·9	116	66·3	237	60·0	692	60·7
Fast	111	28·1	57	32·6	45	25·7	123	31·1	336	29·5
Sedentary time						^*^		^***^		
<3 h	189	47·8	81	46·3	66	37·7	136	34·4	472	41·4
3–5 h	167	42·3	69	39·4	77	44·0	163	41·3	476	41·8
>=5 h	39	9·9	25	14·3	32	18·3	96	24·3	192	16·8
Weight measurement						^***^		^***^		
Not measured	171	43·3	68	38·9	49	28·0	121	30·6	409	35·9
About once a month	121	30·6	55	31·4	56	32·0	123	31·1	355	31·1
2–3 d/week	74	18·7	35	20·0	42	24·0	104	26·3	255	22·4
Every day	29	7·3	17	9·7	28	16·0	47	11·9	121	10·6
Time between dinner and sleep										
>=2 h	217	54·9	105	60·0	100	57·1	195	49·4	617	54·1
Antidiabetic medicine		^*^						^*^		
Without medication^†^	391	99·0	168	96·0	169	96·6	380	96·2	1108	97·2
Taking medication	4	1·0	7	4·0	6	3·4	15	3·8	32	2·8
Antihypertensive medicine								^***^		
Without medication^†^	373	94·4	160	91·4	160	91·4	345	87·3	1038	91·1
Taking medication	22	5·6	15	8·6	15	8·6	50	12·7	102	8·9
Antilipidemic medicine								^*^		
Without medication^†^	375	94·9	164	93·7	159	90·9	357	90·4	1055	92·5
Taking medication	20	5·1	11	6·3	16	9·1	38	9·6	85	7·5
Socio-economic factors										
Occupation						^***^		^***^		
Factory worker	170	97·1	382	96·7	349	88·4	154	88·0	1055	92·5
Others	5	2·9	13	3·3	46	11·6	21	12·0	85	7·5
Positions in the company						^***^		^***^		
Ordinary employee	235	59·5	94	53·7	78	44·6	163	41·3	570	50·0
With positions	160	40·5	81	46·3	97	55·4	232	58·7	570	50·0
Work shift						^***^		^***^		
Daytime worker	68	17·2	36	20·6	76	43·4	163	41·3	343	30·1
Shift worker	327	82·8	139	79·4	99	56·6	232	58·7	797	69·9
Working hours						^***^		^***^		
=<8 h	273	69·1	119	68·0	93	53·1	210	53·2	695	61·0
8–9 h	107	27·1	48	27·4	56	32·0	138	34·9	349	30·6
>=9 h	15	3·8	8	4·6	26	14·9	47	11·9	96	8·4

VL_SL, low vegetables intake and low salt intake; VL_SH, low vegetables intake and high salt intake; VH_SL, high vegetables intake and low salt intake; VH_SH, high vegetables intake and high salt intake.

* *P* < 0·05.

** *P* < 0·01.

*** *P* < 0·001; generalised linear model (binomial or Poisson distribution).

† The without medication group includes those with no records of hospital visits (*n* 323).

### Dietary, anthropometric and blood test and lifestyle and socio-economic factors associated with vegetables and/or salt intake

Examining the associations between vegetables and salt intake and various dietary factors revealed similar patterns for both (see online supplementary material, Supplemental Fig. S2). However, vegetables intake was positively associated with consuming a healthy meal set intake but negatively associated with cereals and meats intake, whereas salt intake was positively associated with eggs intake but negatively associated with fats and oils intake.

Online supplementary material, Supplemental Fig. S3, shows the association between vegetables and salt intake, anthropometric and blood test measurements and lifestyle and socio-economic factors. For each vegetables and salt intake group, the associations with anthropometric and blood test factors were generally consistent. The opposite association between vegetables and salt intake was found for TAG, whereas HDL was negatively associated only with salt intake. Vegetables intake was associated with numerous lifestyle factors unlike salt intake.

### Changes in cardiometabolic measures according to vegetables–salt groups

Table 4 shows the results of the robust regression estimation of the change in cardiometabolic measures among the four vegetables–salt groups with reference to the VL_SH group. An inverse marginal association with change in TAG levels was observed (*β*: –0·11, 95 % CI: –0·23, 0·01, *P*: 0·065 in Model 2; *β*: –0·09, 95 % CI: –0·21, 0·02, *P*: 0·081 in Model 3) in the VH_SL group.

**Table 4.** Estimated change in cardiometabolic measures according to the vegetables/salt intake group^†^

	Model 1			Model 2			Model 3		
	*β*	95 % CI	*P* value	*β*	95 % CI	*P* value	*β*	95 % CI	*P* value
Change in BMI (kg/m^2^)									
VL_SL (*n* 395)	0·07	–0·07, 0·22	0·333	0·07	–0·08, 0·21	0·378	0·06	–0·08, 0·21	0·394
VH_SL (*n* 175)	0·03	–0·14, 0·20	0·720	0·03	–0·14, 0·21	0·705	0·04	–0·14, 0·21	0·670
VH_SH (*n* 395)	0·03	–0·12, 0·17	0·723	–0·002	–0·15, 0·15	0·976	0·01	–0·14, 0·15	0·929
Change in systolic blood pressure (mmHg)									
VL_SL (*n* 395)	–0·55	–2·63, 1·53	0·603	–0·50	–3·01, 2·00	0·558	–0·51	–2·68, 1·66	0·387
VH_SL (*n* 175)	0·20	–2·24, 2·63	0·873	0·09	–2·63, 2·81	0·993	0·96	–1·40, 3·32	0·504
VH_SH (*n* 395)	–0·33	–2·42, 1·75	0·754	–0·53	–2·68, 1·63	0·648	–0·34	–2·21, 1·53	0·775
Change in diastolic blood pressure (mmHg)									
VL_SL (*n* 395)	–0·30	–1·85, 1·25	0·702	–0·53	–2·39, 1·33	0·607	–0·19	–1·91, 1·53	0·984
VH_SL (*n* 175)	–0·70	–2·52, 1·12	0·449	–1·18	–3·20, 0·84	0·247	–0·41	–2·28, 1·46	0·755
VH_SH (*n* 395)	–0·39	–1·95, 1·16	0·622	–0·77	–2·37, 0·83	0·312	–0·02	–1·50, 1·46	0·916
Change in LDL-cholesterol (mmol/L)									
VL_SL (*n* 395)	0·01	–0·08, 0·11	0·780	0·01	–0·08, 0·11	0·779	0·008	–0·08, 0·10	0·875
VH_SL (*n* 175)	–0·08	–0·19, 0·03	0·151	–0·07	–0·18, 0·04	0·213	–0·06	–0·17, 0·05	0·251
VH_SH (*n* 395)	–0·04	–0·14, 0·05	0·346	–0·04	–0·14, 0·05	0·400	–0·04	–0·13, 0·05	0·360
Change in HDL-cholesterol (mmol/L)									
VL_SL (*n* 395)	–0·01	–0·05, 0·03	0·486	–0·01	–0·05, 0·03	0·593	–0·01	–0·05, 0·03	0·521
VH_SL (*n* 175)	–0·03	–0·08, 0·01	0·165	–0·03	–0·07, 0·02	0·275	–0·02	–0·07, 0·02	0·367
VH_SH (*n* 395)	–0·01	–0·05, 0·03	0·621	0·0003	–0·04, 0·04	0·989	–0·01	–0·04, 0·03	0·796
Change in TAG (mmol/L)									
VL_SL (*n* 395)	–0·05	–0·16, 0·05	0·309	–0·06	–0·16, 0·04	0·244	–0·06	–0·15, 0·04	0·231
VH_SL (*n* 175)	–0·09	–0·21, 0·03	0·127	–0·11	–0·23, 0·01	0·065	–0·09	–0·21, 0·02	0·081
VH_SH (*n* 395)	–0·03	–0·13, 0·07	0·579	–0·05	–0·15, 0·05	0·330	–0·07	–0·16, 0·03	0·178
Change in Hb A1c (%)									
VL_SL (*n* 395)	–0·02	–0·06, 0·03	0·442	–0·02	–0·06, 0·03	0·440	–0·002	–0·04, 0·04	0·926
VH_SL (*n* 175)	0·001	–0·05, 0·05	0·985	–0·001	–0·05, 0·05	0·966	0·01	–0·03, 0·06	0·570
VH_SH (*n* 395)	–0·02	–0·06, 0·02	0·345	–0·02	–0·06, 0·02	0·299	–0·004	–0·04, 0·04	0·849

Robust regression with MM-estimation using the low vegetables and high salt intake group as reference.

Model 1: Adjusted for age, sex and BMI at baseline.

Model 2: Adjusted for exercise, alcohol, smoking, total energy intake, frequency of cafeteria visit and medication (antihypertensive medicine for systolic and diastolic blood pressure, antilipidemic medicine for LDL-cholesterol and HDL-cholesterol and TAG and antidiabetic medicine for Hb A1c) in addition to variables included in Model 1.

Model 3: Adjusted for baseline variables in addition to those included in Model 2.

VL_SL, low vegetables intake and low salt intake; VH_SL, high vegetables intake and low salt intake; VH_SH, high vegetables intake and high salt intake.

† Reference group: low vegetables and high salt intake (VL_SH, *n* 175).

The results of the sensitivity analyses with limited participants for analysis are shown in online supplementary material, Supplemental Table S1. Restriction of the analysis to men only (*n* 1089) revealed that the marginal association between TAG change and the VH_SL group (reference: VL_SH; *β*: –0·12, 95 % CI: –0·24, 0·002, *P*: 0·054 in Model 2; *β*: –0·10, 95 % CI: –0·21, 0·01, *P*: 0·085 in Model 3) remained unchanged. Furthermore, the analysis of workers who used the cafeteria for >=18 d (*n* 1027) and those who used it for >= 71 d (*n* 963) revealed strengthening of the association between TAG change and the VH_SL group (reference group: VL_SH; *β*: –0·16, 95 % CI: –0·29, –0·03, *P*: 0·018 in Model 2; *β*: –0·13, 95 % CI: –0·26, –0·01, *P*: 0·042 in Model 3 for >= 18 d; *β*: –0·15, 95 % CI: –0·29, –0·02, *P*: 0·027 in Model 2; and *β*: –0·13, 95 % CI: –0·26, –0·01, *P*: 0·042 in Model 3 for >= 71 d).

The results of the sensitivity analyses for Model 2 with further adjustment variables are shown in the online supplementary material, Supplemental Table S2. When further dietary habits were added, the marginal association between the change in TAG levels and the VH_SL group with reference to the VL_SH group remained unchanged (*β*: –0·10, 95 % CI: –0·23, 0·02, *P*: 0·056); when further lifestyle and socio-economic factors (*β*: –0·08, 95 % CI: –0·20, 0·04, *P*: 0·176) or both lifestyle and socio-economic factors and dietary habit (*β*: –0·10, 95 % CI: –0·22, 0·03, *P*: 0·174) were added, the association was attenuated.

## Discussion

This study objectively examined the dietary choices of workers in workplace cafeterias using data from an automated payment system that an integrated-circuit chip–embedded tableware and worker identification. Vegetables and salt intake alongside user characteristics such as anthropometric measurements and lifestyle and socio-economic factors were analysed. Regarding the association between vegetables and salt intake and cardiometabolic health, an inverse marginal association was observed in the change in TAG levels among individuals with high vegetables intake and low salt intake compared with those with low vegetables intake and high salt intake.

Herein, a habitually higher vegetables and lower salt intake in a workplace cafeteria were more likely to be associated with negative changes in serum TAG levels compared with that of the group with lower vegetables intake and higher salt intake. Existing evidence on the association between the consumption of vegetables (and occasionally fruits) and TAG levels is inconclusive^(8,28–31)^. There have been reports of no association when only the intake of vegetables is evaluated^(28,31)^, and conversely, an inverse association with changes in TAG levels has been reported for soluble fibre intake^(28)^. A recent meta-analysis of randomised controlled trials reported that daily vegetable and fruit intake contributes to significant decreases in serum TAG levels, further reporting that favourable dietary changes other than vegetable and fruit yielded even more favourable changes, particularly in serum TAG levels^(8)^. Conversely, a recent meta-analysis of randomised controlled trials reported that Na reduction increases TAG levels^(32)^. However, the meta-analysis reported a daily Na intake reduction of about 8 g (merely 2·7 g/meal) which far exceeded the difference of <1 g observed between the high and low salt groups in the current study. Furthermore, it is possible that workers who consumed more vegetables and less salt in the current study had healthy dietary habits outside the workplace cafeteria that benefited their cardiometabolic health. Herein, vegetables intake was associated with lifestyle habits, suggesting that other unmeasured lifestyle factors support cardiometabolic health in the participants with higher vegetables intake. Thus, the inverse association observed between vegetables intake in the workplace cafeteria and changes in TAG levels could potentially be attributed to unmeasured lifestyle factors related to vegetables intake.

Although meta-analyses of randomised controlled trials have reported that vegetables intake has a favourable effect on blood pressure^(7,8,30)^, no apparent association was observed in the present study. The impact of vegetables intake on LDL-C and HDL-C, similar to that on TAG, remains inconclusive. Therefore, further studies involving different populations and samples are warranted on the association between vegetables and salt intake in workplace cafeterias and cardiometabolic health.

Interestingly, while the associations between vegetables and salt intake and various dietary factors were similar, vegetables intake was associated with lifestyle, but salt intake was not. This suggests that choosing a menu that contains vegetables in the workplace cafeteria is one of the practices of a healthy lifestyle, such as exercising, not drinking, not smoking and measuring one’s weight. Currently, reducing salt intake might not be considered a practice for a healthy lifestyle, representing a lack of awareness regarding the association between salt intake and health. Therefore, workplace health promotion strategies should aim to increase awareness regarding the importance of reducing salt intake as part of a healthy lifestyle and also offer vegetable-rich and low-salt meals such as healthy meal sets designed by registered dietitians in workplace cafeterias.

Herein, the group with higher vegetables intake were older, exhibited unfavourable baseline cardiometabolic measures and were at increased cardiometabolic risk. It is possible that some individuals who exhibited unfavourable results during the baseline health examination may have opted for higher vegetables intake as part of their healthy lifestyle practices. This may have contributed to the comparatively worse baseline cardiometabolic measures observed in this group.

Regarding the models in the present study, since the adjustment of the baseline measures of outcomes is controversial^(33)^, we used three statistical models for the adjustment. In Model 3, we considered each baseline value of the dependent variable in addition to the variables adjusted in Model 2. However, as this could potentially lead to over-adjustment owing to the influence of the independent variables (vegetables and salt) and other relevant factors, Model 2 was considered more suitable.

Regarding the sensitivity analysis, the association may have been strengthened when the individuals were limited to those who visited the cafeteria for ≥71 d as their long-term, continuous eating habits in the workplace cafeteria could be assessed with greater accuracy. After adjusting for all socio-economic factors, the association between vegetables and salt intake and the change in TAG levels were attenuated, with only shift work being statistically significantly associated with TAG among the additional socio-economic factors in this model. In this regard, shift workers reportedly take dinner at irregular times, leading to an imbalance of foods and nutrients in their diets^(34)^. Thus, shift work may be a proxy indicator of a food/nutrient imbalance in daily diet, and adjustment at the same time as vegetables intake may be an over-adjustment for the association with changes in TAG levels in the present study.

The strength of this study lies in the fact that the meals of the workers were objectively evaluated without their awareness and with the same burden as their regular eating in the workplace cafeteria. In practice, dietary surveys equivalent to the dietary record method were conducted in the workplace cafeteria over a long period of time without subject burden and with minimal subjective bias. Furthermore, cardiometabolic measurements, lifestyle habits and other worker characteristics were investigated during normal health examinations by medical staff unrelated to the dietary survey. Thus, a major strength of the data in the present study is that it minimises bias and measurement error related to investigations of observational studies.

This study has some limitations. First, we could not assess meals other than those served in the workplace cafeteria. However, vegetables intake is associated with lifestyle habits and is likely to represent the daily dietary habits of the workers. Conversely, salt intake is not likely to represent meals consumed outside of the workplace cafeteria. In addition, condiments are freely available to be added at the food selection and dining area. As with other dietary surveys, the possibility cannot be ruled out that there was an error of some magnitude in the estimated amount of salt intake. However, herein, nutrients were estimated from the actual menu information (ingredients and seasonings) provided, which allowed for an objective assessment of the approximate salt intake in the selected menu items. Second, a causal relationship cannot be inferred. Because exposure from eating in the cafeteria has been ongoing since the beginning of the workday, it is difficult to adjust for prebaseline confounding. In addition, important confounding factors have been adjusted, although we cannot rule out the possibility that unmeasured or not proxied confounders can still contribute to the associations in the present study. Third, the amount of leftover food was not assessed. Herein, the amount of food served was defined as the amount of food consumed. However, the association is expected to shift towards attenuated when there is leftover food. Fourth, the number of days of the dietary survey (i.e. the number of times the cafeteria was used) varied among the participants. Therefore, there was variation among individuals in the accuracy of the dietary survey. However, as the present study included the number of times the cafeteria was used in the model and the sensitivity analysis was limited to those who used the cafeteria for ≥71 d, we believe that the impact of this issue is small.

In conclusion, the current study objectively assessed the real-life eating habits of employees using the workplace cafeteria and found an inverse change in blood TAG levels among those with higher vegetables and lower salt intake compared with those with lower vegetables and higher salt intake. These findings show that diet plays an important role in occupational health and emphasise the importance of collecting basic data for the purpose of promoting health through workplace cafeteria meals.

## Supporting information

Shirai et al. supplementary material 1 Shirai et al. supplementary material

Shirai et al. supplementary material 2 Shirai et al. supplementary material

Shirai et al. supplementary material 3 Shirai et al. supplementary material

Shirai et al. supplementary material 4 Shirai et al. supplementary material

Shirai et al. supplementary material 5 Shirai et al. supplementary material

Shirai et al. supplementary material 6 Shirai et al. supplementary material

## Data Availability

The original data used in this study are unavailable to the public as per the policy of Toyota Motor Corporation.

## Supplementary material

For supplementary material accompanying this paper, visit https://doi.org/10.1017/S1368980024001162
