# Supplementary figures and images for: Characteristics of and changes in the cardiometabolic measures of Japanese workers grouped according to their vegetables and salt intake through workplace cafeteria meals
Source: Public Health Nutr. 2024 May 24;27(1):e155. doi: 10.1017/S1368980024001162 (PMC11626606; doi:10.1017/S1368980024001162)

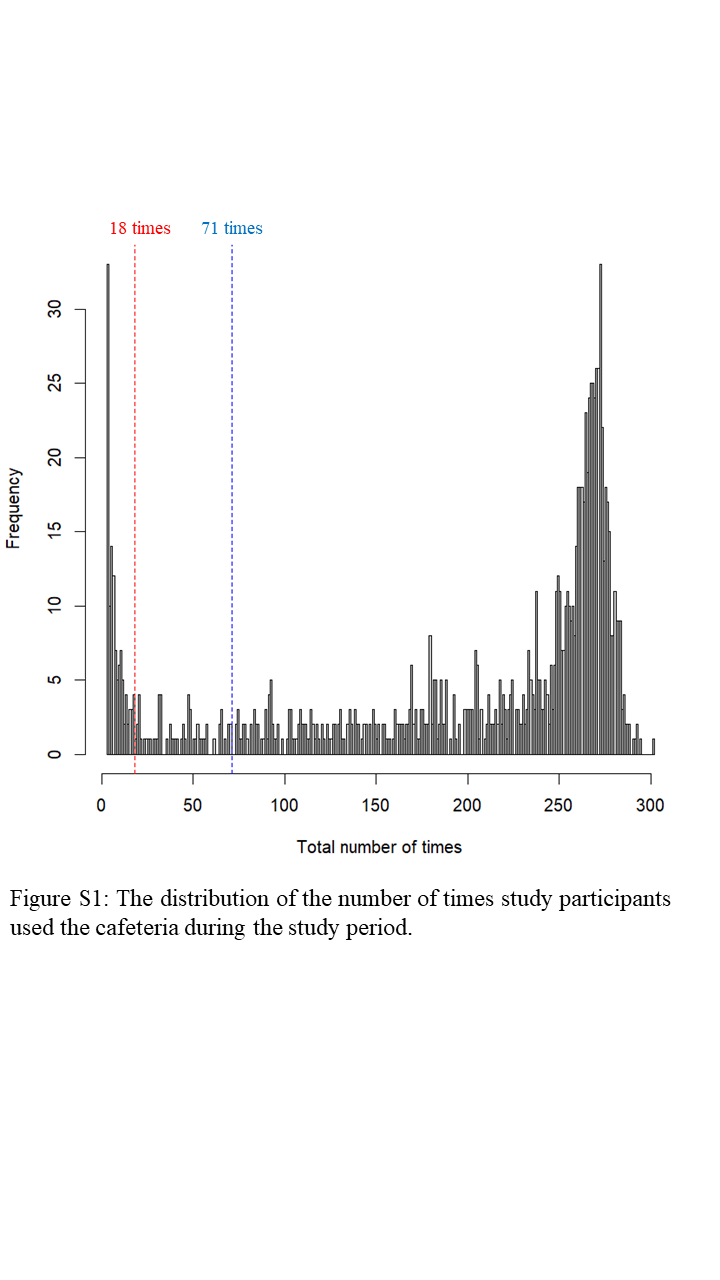

Supplement: Shirai et al. supplementary material 1 — Shirai et al. supplementary material [file S1368980024001162sup001.tif]

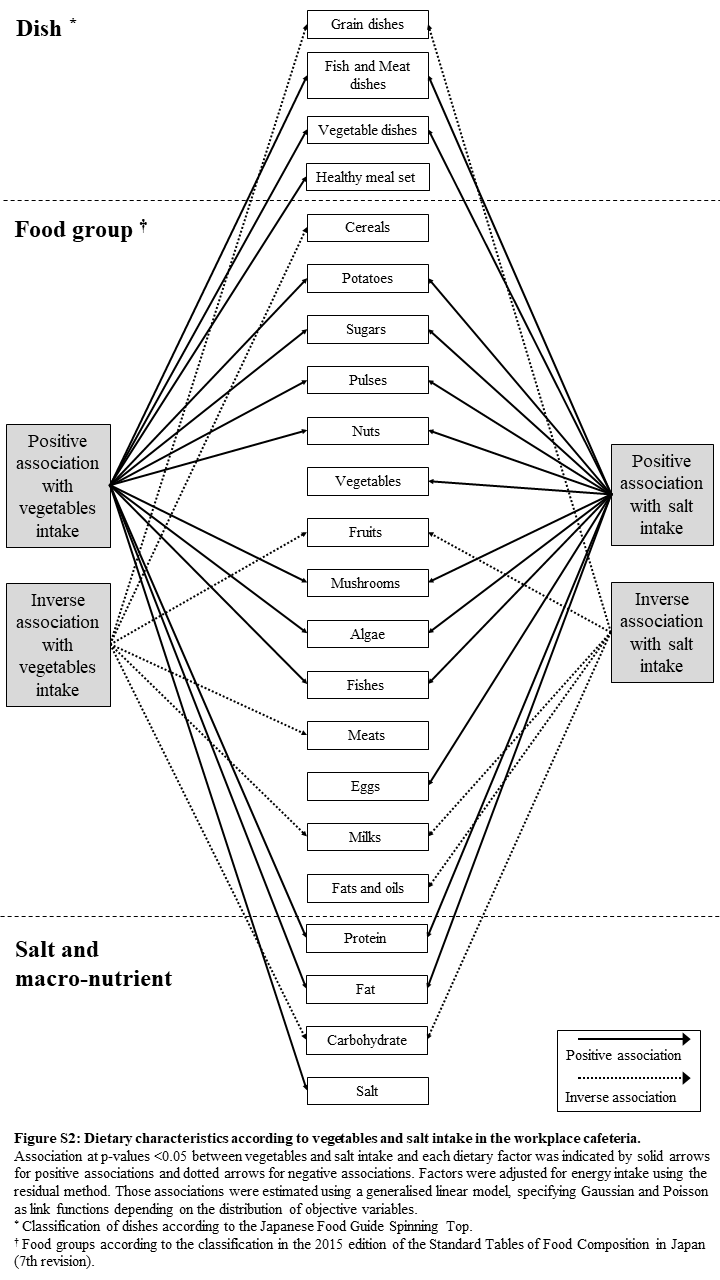

Supplement: Shirai et al. supplementary material 2 — Shirai et al. supplementary material [file S1368980024001162sup002.tif]

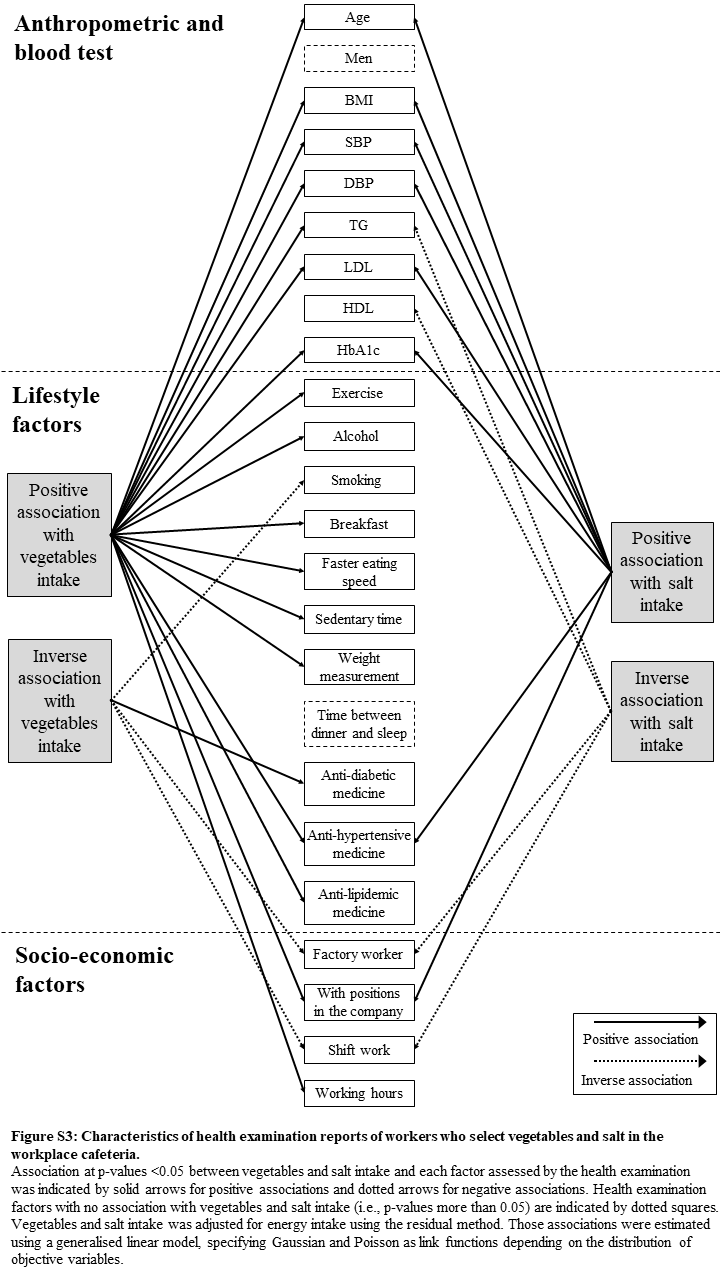

Supplement: Shirai et al. supplementary material 3 — Shirai et al. supplementary material [file S1368980024001162sup003.tif]
