# Supplementary material for: Characteristics of and changes in the cardiometabolic measures of Japanese workers grouped according to their vegetables and salt intake through workplace cafeteria meals
Source: Public Health Nutr. 2024 May 24;27(1):e155. doi: 10.1017/S1368980024001162 (PMC11626606; doi:10.1017/S1368980024001162)
Supplement: Shirai et al. supplementary material 4 — Shirai et al. supplementary material [file S1368980024001162sup004.pdf]

Table S1. Examples of the food composition of dishes actually served in the cafeteria during the study period.

Dish*	Food	Amount
Grain dish (1):	Raw rice	150g
"Curry Rice" †	Beef curry	230g
	pickles	20g
Grain dish (2):	Japanese Soba Noodles	200g
"Zaru Soba" †	Soba-Soup	30mL
	Ground sesame (white)	1g
	Wasabi (Japanese horseradish)	2g
	Seaweed (Nori)	1g
	Spring onion	20g
Grain dish (3):	Ramen noodles	200g
"Sapporo Ramen" †	Pork belly	3 pcs
	Green bean sprouts	30g
	Onion	40g
	Whole corn	15g
	Miso ramen soup	40mL
	Chicken soup powder	1g
	Dried seaweed (Wakame)	1g
	Spring onion	15g
Fish and Meat dish (1)	Cabbage	40g
"Tori no Sanzokuyaki" † (chicken steak)	Dressing	15mL
	Pasta	15g
	Salt	1g
	Pepper	0.50g
	Linoleic salad oil	1g
	Chicken thigh	160g
	Sauce	15g
Fish and Meat dish (2)	flounder (esp. the Japanese flounder)	1 pcs (approx. 140g)
" Karei no Nitsuke" † (braised karei)	Soy sauce	15mL
	Mirin (sweet cooking sake)	4mL
	Sugar	8g
	Ginger	2g
	green beans	20g
Fish and Meat dish (3)	Tofu	0.5 pcs (approx. 150g)
"Yakko Dofu" † (fresh tofu)	Ginger	5g
	Spring onion	5g
	Dried bonito flakes	0.50g
	Soy sauce	10mL
Vegetable dish (1)	Lettuce	6g
"Nama Yasai Sarada" † (raw vegetable salad)	Cabbage	30g
	Cucumber	15g
	Cherry tomatoes	1 pcs

	Whole corn	10g
	Dressing	15mL
Vegetable dish (2)	Radish	80g
"Daikon no Pirikarani" † (spicy radish stew)	Minced chicken	20g
	Scallions	3g
	Linoleic salad oil	1.50g
	Grated garlic	1g
	Bean-paste sauce	0.50g
	soy sauce	7mL
	Sugar	3g
	Cooking liquor	5mL
	Sesame oil	1g
Vegetable dish (3)	Konjac	120g
"Hirihiri Konnyaku Ni" † (spicy konjac stew)	Green beans	20g
	soy sauce	12mL
	Sugar	6g
	Sesame oil	3g
	Hot pepper	0.20g

* Grain dishes (Syushoku: Staple dishes), including rice, bread, noodles, and pasta, in which the main ingredient is a source of carbohydrate; Fish and Meat dishes (Syusai: Main dishes), including meat, fish, egg and soy-bean dishes, in which the main ingredient is a source of protein; Vegetable dishes (Fukusai: Side dishes), including vegetables, potatoes, pulses (excluding soya), mushrooms and algae, in which the main ingredient is a source of various vitamins, minerals and dietary fibre.

† Name of dish in Japanese.
