# Supplementary material for: Characteristics of and changes in the cardiometabolic measures of Japanese workers grouped according to their vegetables and salt intake through workplace cafeteria meals
Source: Public Health Nutr. 2024 May 24;27(1):e155. doi: 10.1017/S1368980024001162 (PMC11626606; doi:10.1017/S1368980024001162)
Supplement: Shirai et al. supplementary material 5 — Shirai et al. supplementary material [file S1368980024001162sup005.pdf]

Table S2. Estimated change in serum lipids for three groups with high vegetable and/or low salt intake, based on a reference group with low vegetable and high salt intake in the subgroup sensitivity analyses.

	Model 1			Model 2			Model 3		
	β	95% CI	p-value	β	95% CI	p-value	β	95% CI	p-value
**Change in low-density lipoprotein cholesterol (mmol/L)**									
**Male workers (n = 1,089)**									
VL_SL (n = 375)	0.005	−0.105, 0.115	0.754	0.010	−0.101, 0.120	0.769	0.004	−0.105, 0.112	0.865
VH_SL (n = 169)	−0.097	−0.223, 0.029	0.153	−0.072	−0.202, 0.058	0.210	−0.061	−0.189, 0.066	0.260
VH_SH (n = 374)	−0.079	−0.186, 0.029	0.335	−0.071	−0.181, 0.038	0.372	−0.077	−0.185, 0.03	0.340
**Workers with a cafeteria usage record of ≥18 days (n = 1027)**									
VL_SL (n = 353)	0.024	−0.076, 0.124	0.635	0.025	−0.076, 0.125	0.633	0.021	−0.077, 0.119	0.673
VH_SL (n = 150)	−0.082	−0.197, 0.033	0.162	−0.068	−0.186, 0.049	0.254	−0.059	−0.173, 0.056	0.317
VH_SH (n = 368)	−0.042	−0.139, 0.055	0.396	−0.041	−0.139, 0.057	0.415	−0.044	−0.139, 0.053	0.375
**Workers with a cafeteria usage record of ≥71 days (n = 963)**									
VL_SL (n = 304)	0.017	−0.086, 0.119	0.750	0.019	−0.084, 0.122	0.718	0.015	−0.086, 0.116	0.766
VH_SL (n = 127)	−0.072	−0.190, 0.046	0.232	−0.052	−0.173, 0.069	0.400	−0.044	−0.162, 0.075	0.469
VH_SH (n = 297)	−0.058	−0.157, 0.041	0.253	−0.051	−0.153, 0.050	0.323	−0.052	−0.151, 0.048	0.307
**Change in high density lipoprotein cholesterol (mmol/L)**									
**Male workers (n = 1,089)**									
VL_SL (n = 375)	−0.012	−0.052, 0.028	0.558	−0.010	−0.050, 0.031	0.645	−0.011	−0.051, 0.028	0.575
VH_SL (n = 169)	−0.036	−0.082, 0.010	0.125	−0.030	−0.077, 0.017	0.205	−0.024	−0.070, 0.022	0.299
VH_SH (n = 374)	−0.010	−0.049, 0.030	0.620	0.0003	−0.040, 0.040	0.989	−0.006	−0.046, 0.033	0.748
**Workers with a cafeteria usage record ≥18 days (n = 1027)**									
VL_SL (n = 353)	−0.021	−0.063, 0.021	0.333	−0.017	−0.059, 0.025	0.433	−0.020	−0.061, 0.022	0.357
VH_SL (n = 150)	−0.039	−0.088, 0.009	0.111	−0.032	−0.081, 0.017	0.206	−0.027	−0.076, 0.022	0.280
VH_SH (n = 368)	−0.016	−0.056, 0.025	0.452	−0.008	−0.049, 0.034	0.723	−0.012	−0.053, 0.029	0.567
**Workers with a cafeteria usage record ≥71 days (n = 963)**									
VL_SL (n = 304)	−0.022	−0.065, 0.021	0.319	−0.021	−0.064, 0.023	0.351	−0.024	−0.067, 0.019	0.281
VH_SL (n = 127)	−0.052	−0.102, −0.002	0.041	−0.047	−0.098, 0.004	0.073	−0.041	−0.092, 0.010	0.113
VH_SH (n = 297)	−0.027	−0.069, 0.016	0.215	−0.021	−0.064, 0.022	0.338	−0.024	−0.067, 0.018	0.263
**Change in triglycerides (mmol/L)**									
**Male workers (n = 1,089)**									
VL_SL (n = 375)	−0.051	−0.154, 0.053	0.337	−0.058	−0.162, 0.045	0.271	−0.055	−0.152, 0.041	0.262
VH_SL (n = 169)	−0.098	−0.217, 0.021	0.106	−0.118	−0.238, 0.002	0.054	−0.099	−0.211, 0.013	0.085
VH_SH (n = 374)	−0.030	−0.131, 0.072	0.567	−0.053	−0.156, 0.050	0.314	−0.068	−0.164, 0.029	0.169
**Workers with a cafeteria usage record ≥18 days (n = 1027)**									
VL_SL (n = 353)	−0.078	−0.188, 0.033	0.167	−0.087	−0.198, 0.024	0.123	−0.081	−0.184, 0.021	0.121
VH_SL (n = 150)	−0.129	−0.256, −0.001	0.048	−0.157	−0.286, −0.027	0.018	−0.128	−0.248, −0.008	0.037
VH_SH (n = 368)	−0.052	−0.159, 0.055	0.344	−0.067	−0.175, 0.042	0.230	−0.082	−0.183, 0.019	0.110
**Workers with a cafeteria usage record ≥71 days (n = 963)**									
VL_SL (n = 304)	−0.071	−0.186, 0.043	0.222	−0.079	−0.194, 0.036	0.179	−0.070	−0.177, 0.037	0.201
VH_SL (n = 127)	−0.121	−0.253, 0.011	0.073	−0.153	−0.288, −0.018	0.027	−0.131	−0.257, −0.005	0.042
VH_SH (n = 297)	−0.059	−0.170, 0.052	0.301	−0.075	−0.188, 0.038	0.193	−0.088	−0.194, 0.018	0.104

Robust regression with MM-estimation with reference to the group with low vegetable and high salt intake.

Model 1: Age, sex, and body mass index at baseline were adjusted for.

Model 2: Exercise, alcohol, smoking, total energy intake, cafeteria visit, and medication (antihypertensive medicine for systolic and diastolic blood pressure, antilipidemic medicine for low-density and high-density lipoprotein cholesterol and triglycerides, and antidiabetic medicine for hemoglobin A1c) were adjusted in addition to Model 1.

Model 3: In Addition to those in Model 2, objective variables at baseline were adjusted for.

Abbreviations: CI: confidence interval; VL_SL: low vegetable intake and low salt intake; VH_SL: high vegetable intake and low salt intake; VH_SH: high vegetable intake and high salt intake.
