# Supplementary material for: Characteristics of and changes in the cardiometabolic measures of Japanese workers grouped according to their vegetables and salt intake through workplace cafeteria meals
Source: Public Health Nutr. 2024 May 24;27(1):e155. doi: 10.1017/S1368980024001162 (PMC11626606; doi:10.1017/S1368980024001162)
Supplement: Shirai et al. supplementary material 6 — Shirai et al. supplementary material [file S1368980024001162sup006.pdf]

Table S3. Estimated change in serum lipids for the three groups with high vegetable and/or low salt intake based on a reference group with low vegetable and high salt intake in sensitivity analyses

	Change in LDL-C (mmol/L)			Change in HDL-C (mmol/L)			Change in triglycerides (mmol/L)		
	β	95% CI	p value	β	95% CI	p value	β	95% CI	p value
**Added variables on lifestyle and socioeconomic factors* to Model 2**									
VL_SL	0.010	−0.085, 0.106	0.832	−0.010	−0.051, 0.031	0.632	−0.064	−0.167, 0.040	0.230
VH_SL	−0.070	−0.183, 0.042	0.217	−0.029	−0.077, 0.018	0.228	−0.084	−0.205, 0.038	0.176
VH_SH	−0.042	−0.139, 0.054	0.387	−0.002	−0.043, 0.039	0.917	−0.027	−0.131, 0.078	0.615
**Added variables on dietary habit† to Model 2**									
VL_SL	0.010	−0.087, 0.107	0.822	−0.013	−0.054, 0.028	0.549	−0.059	−0.164, 0.046	0.305
VH_SL	−0.073	−0.185, 0.038	0.194	−0.001	−0.041, 0.039	0.350	−0.105	−0.225, 0.016	0.056
VH_SH	−0.043	−0.139, 0.053	0.382	−0.028	−0.075, 0.018	0.926	−0.040	−0.145, 0.064	0.334
**Added variables on lifestyle and socioeconomic factors, and dietary habit to Model 2**									
VL_SL	0.008	−0.089, 0.105	0.876	−0.010	−0.051, 0.031	0.596	−0.068	−0.173, 0.037	0.313
VH_SL	−0.080	−0.192, 0.033	0.201	−0.001	−0.042, 0.040	0.292	−0.096	−0.217, 0.025	0.174
VH_SH	−0.049	−0.146, 0.048	0.375	−0.026	−0.073, 0.021	0.981	−0.029	−0.134, 0.075	0.688

Robust regression with MM-estimation was used with reference to the group of low vegetables and high salt intake.
Abbreviations: CI: confidence interval; HDL-C: high-density lipoprotein cholesterol; LDL-C: low-density lipoprotein cholesterol; VL_SL: low vegetables intake and low salt intake; VH_SL: high vegetables intake and low salt intake; VH_SH: high vegetables Intake and high salt intake.

* Sedentary time, weight measurement, occupation, positions in the company, work shift, and working hours

† Breakfast, subjective eating speed, and time between dinner and sleep
